# Rare Association Between Lichen Planus and Vogt-Koyanagi-Harada Syndrome: A Case Report

**DOI:** 10.7759/cureus.35464

**Published:** 2023-02-25

**Authors:** Mohammed Al-Haddab, Sahar Alsharif, Turky Alsehli, Feras Altukhaim

**Affiliations:** 1 Dermatology, King Saud University, Riyadh, SAU

**Keywords:** dermatology case report, autoimmune disorder, vkh syndrome, vogt–koyanagi–harada syndrome, lichen planus

## Abstract

Vogt-Koyanagi-Harada syndrome (VKHS) is a rare inflammatory autoimmune condition that affects tissues with high melanocyte concentrations such as the skin, inner ears, eyes, and central nervous system. VKHS has previously been reported to be associated with various autoimmune diseases, but we have observed the first known co-occurrence of VKHS and lichen planus (LP). In this report, we examine the similarities between these two diseases and discuss how they relate to each other.

## Introduction

Vogt-Koyanagi-Harada syndrome (VKHS) is a granulomatous autoimmune disorder that usually affects tissues with high melanocyte concentrations such as the skin, inner ears, eyes, and central nervous system. VKHS is characterized by cutaneous signs like alopecia, vitiligo, and poliosis; ocular signs like bilateral uveitis, papillitis, and serous (exudative) retinal detachment; and neuroauditory signs like meningismus, cranial nerve palsies, deafness, tinnitus, and vertigo [1,2].

Lichen planus (LP) is a chronic autoimmune disease that affects the skin, mucosal surfaces, hair, and nails. Cutaneous lichen planus (CLP) typically presents with violaceous papules and plaques that may be itchy, shiny, and covered by fine white lines (Wickham striae). The lesions can occur anywhere but appear most commonly on the wrists, lower back, and ankles [3,4]. 

Although both VKHS and LP are autoimmune diseases, no combined VKHS and LP cases have previously been reported. In this case, we encountered a patient who was known to have VKHS and presented with-new onset LP.

## Case presentation

A 50-year-old Saudi female with VKHS was referred to our dermatology clinic by an ophthalmologist. At her initial presentation, the patient exhibited severe eye redness and pain, which were secondary to acute uveitis. She was treated with prednisolone and mycophenolate mofetil and experienced significant improvement in her ocular symptoms.

The patient presented to us with poliosis of the left temporal scalp and widespread depigmented patches of skin across the left leg, right side of the chest, and right eyebrow (without alopecia). According to her clinical presentation, and in correlation with her primary disease of VKHS, we made a diagnosis of vitiligo. We prescribed treatment with the twice-daily administration of tacrolimus 0.1% ointment, which markedly improved the patient’s condition.

**Figure 1:**
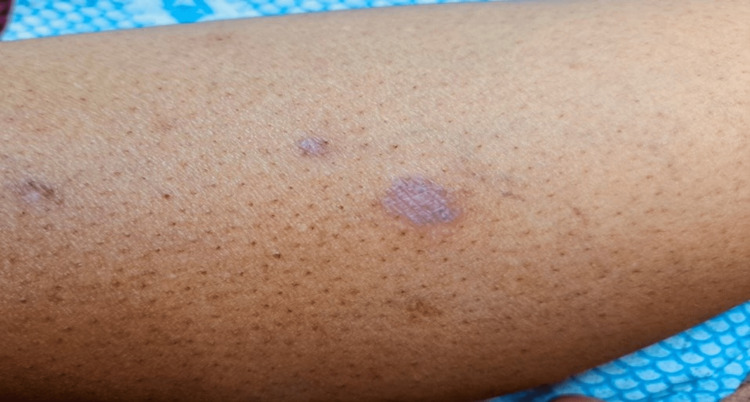
Multiple 1 to 3 cm violaceous flat-topped plaques and papules with a lattice-like network of white lines (Wickham striae) on the leg

After two years, the patient presented again to our dermatology clinic, complaining of an itchy skin rash that had been affecting both of her legs for one month. She denied any recent history of infections, traumas, or new drug use. No family history of dermatological disorders. On physical examination, we noted multiple flat-topped violaceous plaques and papules covered with a lattice-like network of white lines on the legs and feet (Figure 1).

**Figure 2:**
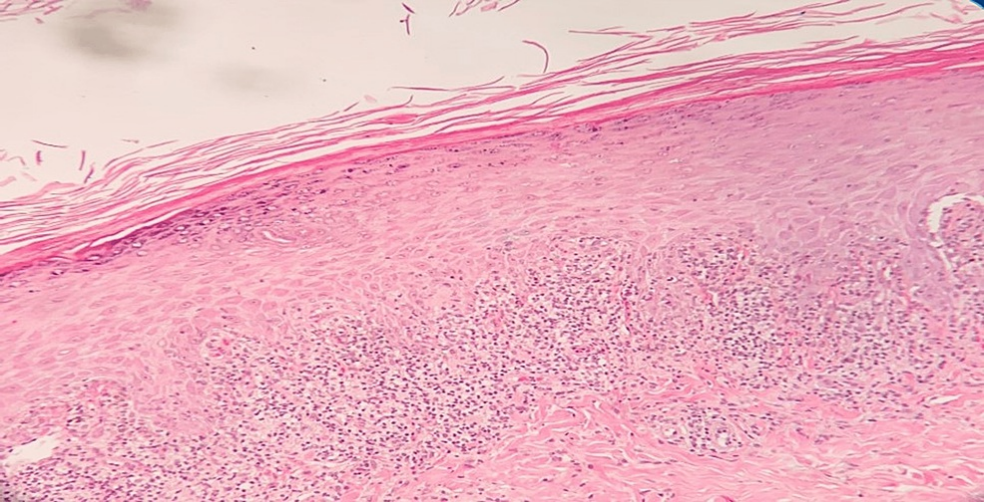
Skin biopsy from the leg showed hyperkeratosis, acanthosis, hypergranulosis, sawtooth appearance of rete ridges, and lichenoid interface dermatitis

A skin biopsy revealed hyperkeratosis, acanthosis, hypergranulosis, sawtooth-shaped rete ridges, and lichenoid interface dermatitis (Figure 2).

We made a diagnosis of lichen planus based on clinical and histopathological findings. The patient was prescribed betamethasone dipropionate 0.05% ointment, twice daily, and exhibited a positive response to treatment.

## Discussion

VKHS is a multisystemic granulomatous autoimmune disease with integumentary, central nervous system, auditory, and ocular manifestations. This syndrome has four clinically defined stages. It begins with the prodromal stage, which is characterized by neuroauditory signs. Then follows the acute uveitic stage, during which ocular symptoms occur. Next comes the convalescent stage, which is characterized by the presence of dermatological manifestations. Finally, the disease progresses to the chronic recurrent stage, which is characterized by recurrent inflammations of the anterior uvea and ophthalmological complications [1].

The American Uveitis Society’s diagnostic criteria for VKHS include no history of ocular trauma (including surgery) and at least one finding in three of the following four categories: (a) bilateral chronic iridocyclitis; (b) posterior uveitis, including exudative retinal detachment, disc hyperemia or edema, and sunset glow fundus; (c) neurological signs, such as tinnitus and meningismus; (d) cutaneous evidence of alopecia, poliosis, or vitiligo (Nicula & Szabo, 2016) [5].

The exact pathogenesis behind VKHS is still unknown. A recent theory suggests that some viral infections in VKHS patients may induce T-cell-mediated immunity against their bodies’ melanocytes [6]. Notably, the etiopathogenesis of LP also involves an immune attack by CD4 and CD8 T-cells on basal keratinocytes [7]. Moreover, both diseases are strongly associated with human leukocyte antigens (HLAs). Recent genetic studies have implicated the presence of HLA DR1 in some patients with VKHS and LP. In addition, both diseases correlate with immunological abnormalities and genetic predispositions such as familial occurrence [8,9]. Both disorders are also well-known to be associated with other autoimmune disorders such as Hashimoto’s thyroiditis [3,10]. To the best of our knowledge, our patient represents the first reported case of concurrent VKHS and LP. It is possible that this co-occurrence was coincidental; however, further studies should be performed to investigate whether these diseases are related.

## Conclusions

VKHS is a rare autoimmune syndrome that affects the skin and other organs. We observed the first known co-occurrence of VKHS and LP. Although this association could be incidental, the diseases share multiple similarities that may link them together. More research into their relationship should be done in the future.

## References

[1] Mehta KN, Daigavane S (2022). Case of probable Vogt-Koyanagi-Harada syndrome: a rare presentation. Indian J Ophthalmol.

[2] Shivaram S, Nagappa M, Seshagiri DV (2021). Vogt-Koyanagi-Harada syndrome - a neurologist’s perspective. Ann Indian Acad Neurol.

[3] Gorouhi F, Davari P, Fazel N (2014). Cutaneous and mucosal lichen planus: a comprehensive review of clinical subtypes, risk factors, diagnosis, and prognosis. ScientificWorldJournal.

[4] Boch K, Langan EA, Kridin K, Zillikens D, Ludwig RJ, Bieber K (2021). Lichen Planus. Front Med (Lausanne).

[5] Nicula C, Szabo I (2016). Vogt-Koyanagi-Harada syndrome case report. Rom J Ophthalmol.

[6] Stern EM, Nataneli N (2022). Vogt Koyanagi Harada syndrome. https://www.ncbi.nlm.nih.gov/books/NBK574571/.

[7] Daye M, Temiz SA, Isık B (2021). The relationship between lichen planus and metabolic syndrome. J Cosmet Dermatol.

[8] Le Cleach L, Chosidow O (2012). Clinical practice. Lichen planus. N Engl J Med.

[9] Zhang Q, Fan X, Tian M, Han H (2020). A case presentation of an IgA nephropathy patient with Vogt-Koyanagi-Harada syndrome. BMC Nephrol.

[10] Abdelghani K, Boussaa H, Lajmi H (2021). Rare association between rheumatoid arthritis and Vogt-Koyanagi-Harada syndrome: a case-based review. Egypt. Rheumatol.

